# Supplementary material for: Learning curve and surgical outcome of robotic assisted colorectal surgery with ERAS program
Source: Sci Rep. 2022 Nov 29;12:20566. doi: 10.1038/s41598-022-24665-w (PMC9709162; doi:10.1038/s41598-022-24665-w)
Supplement: Supplementary file 1 — Supplementary Information 1. [file 41598_2022_24665_MOESM1_ESM.zip › LSC Raw data-Ñ[▒K/137/H_ñw╝╨░O▒Kñσ.pdf]

正式摘要(電子簽章：000000226198769)<br>W52 / W52 ☐ 英文標題(注意:無英文姓名-請補病人基本檔!)	☐ 【讀取】憑證	

臺中榮民總醫院

出院病歷摘要

轉入號： 轉出號：

[redacted]

(8)流水編號	
(9)入院日期	110/09/15 (2021) 直腸外科 W52 - 091
(10)轉科日期	
(11)出院日期	110/09/22 (2021) 住院天數計 7 日

診斷

- 入院 :<Active problems>
  1. Sigmoid colon cancer
  <Past history>
  1. CAD-TVD (LAD-os nearly-CTO, LCX-M to -OM2 CTO, RCA-D 95% stenosis) s/p CABG 4(LIMA-RA-LAD-DA-PLB; Ao-RSVG-PDA) on 2021/8/6.
  2. Bifascicular block (RBBB+LPFB), with chronic chest pain for 30+ years, LVEF61%, NYHA II
  3. CKD, stage 3
  4. HTN, under regular medication
- 出院 :<Active problems>
  1. Sigmoid colon cancer s/p laparoscopic anterior resection on 2021/09/16
  <Past history>
  1. CAD-TVD (LAD-os nearly-CTO, LCX-M to -OM2 CTO, RCA-D 95% stenosis) s/p CABG 4(LIMA-RA-LAD-DA-PLB; Ao-RSVG-PDA) on 2021/8/6.
  2. Bifascicular block (RBBB+LPFB), with chronic chest pain for 30+ years, LVEF61%, NYHA II
  3. CKD, stage 3
  4. HTN, under regular medication

主訴

- positive stool OB at health check-up three months ago.

病史

- This 67-year-old man has history of
  1. CAD-TVD (LAD-os nearly-CTO, LCX-M to -OM2 CTO, RCA-D 95% stenosis) s/p
  CABG 4(LIMA-RA-LAD-DA-PLB; Ao-RSVG-PDA) on 2021/8/6.
  2. Bifascicular block (RBBB+LPFB), with chronic chest pain for 30+ years, LVEF61%, NYHA II
  3. CKD, stage 3
  4. HTN, under regular medication
  According to medical record, he was found positive stool OB at health check-up and was diagnosed as S-colon adenocarcinoma three months ago. He was suggested operation but he was found coronary artery disease during preoperative survey and then underwent CABG operation on 2021/08/06. He had CVS and CRS OPD follow-up. Under his relative stable status, he was admitted for further surgery.

體檢發現

-
  - General appearance :
    - easy looking , conscious clear , without cardiopulmonary distress
    - Vital signs自動帶入date 體溫(BT) 脈搏(P) 呼吸(R) 血壓(BP) 身高(BH) 體重(BW) BMI
      20210915 36.3℃ 75次/分 18次/分 129/77mmHg 159.1cm 67.5kg 26.7
    - Vital signs: B.P. 129/77 mmHg , P.R. 75 /min , R.R. 18 /min , B.T. 36.3 ℃
  - HEENT :
    - Eyes: no anemic conjunctiva, no icteric sclera, normal EOM
    - ENT: no otorrhea, no rhinorrhea, no erythematous mucosa, no injected tonsil, no deformity
  - Neck: supple, freely movable, trachea midline,no deformity, no engorged jugular vein ,

- no palpable thyroid or lymph node enlargement
  - Chest:
    - Inspection: Grossly normal, symmetric and fully expansion, no accessory muscle used, no operation scar, no spider angiomata, no gynecomastia
    - Palpation: no palpable mass or nodule, no subcutaneous emphysema, no tenderness
    - Ausculation: clear breathing sound
  - Heart:
    - Percussion: normal heart size
    - Ausculation: regular heart beat, normal S1, S2, no gallop, no murmur
  - Abdomen:
    - Inspection: in
      - flat contour, symetrically, no scar, no stria, no dilated veins, no rashes
    - Ausculation: normoactive bowel sound, no abdominal bruits
    - palpation: soft, no muscle guarding or rigidity, no local tenderness, no rebounding pain, no palpable mass, no palpable pulsation
    - Percussion: no shifting dulllness, no hepatomegaly, no splenomagaly
  -

Peripheral pulsation :	CA	BA	RA	FA	PA	PTA	DPA
Right side :	++	++	++	++	++	++	++/+++
Left side :	++	++	++	++	++	++	++/+++

  - Genito-anus: no tenderness or palpable mass lesion or rectal shelf, normal external genitalia, normal anal tone, brown stool

CRS CONDITION

- CEA: 2.62 ng/ml
-
  - Lung

手術日期及方法

- 手術日期及方法20210916 1. Laparoscopic anterior resection
  2. Intraoperative colonoscopy : (-)
  3. High ligation (+) : At IMA root. IMV were also ligated
  4. Take down from the splenic flexure : (-)
  5. EndoGIA (60-4.1) X1 + EEA (29 mm) were used
  6. Proximal cut end: 12m, distal cut end: 3 cm
  7. One 12 Fr. penrose drain was placed into the pelvic area from LLQ trochar site
- 手術結果及發現：
  - 1. A 0.3 x 0.3 cm tumor at S colon area 2. No other gross tumor nor polyps was found 3. There was no obvious visible liver metastasis, no visible peritoneal seeding 4. No obvious ascites was noted 5. Bilateral doughnuts : intact ; Air leak test: Done and no air leak was found

住院治療經過

- After admission, preoperative prepare was done, he underwent laparoscopic anterior resection on 2021/09/16. After surgery, he had regained conscious and tolerated well. NG was removed and he started to try water at POD-2, try liquid diet at POD-4, semi-liquid diet at POD-5. Under his relative stable condition, he was discharged on 09/22.

合併症

- Nil

一般檢查

- 累積報告：

DATE	TIME	ALB	TP	BIL.T	BIL.D	ALKP	AST	ALT	LDH	AMY	LIPASE	NH3	Ferritin	UA
1100915	2005			1.09	0.22		14	14						
1100916	1453	3.8		0.9	0.2		13	13						

DATE	TIME	WBC	RBC	HGB	HCT	MCV	PLT	BLST	NEUT	BAND	SEG	LYM	MON	EOS	BAS
1100915	2001	6860	4.79	13.1	41.1	85.8	343		61.2			27.3	6.1	4.8	0.6
1100916	1452	10440	4.53	12.6	38.5	85.0	317		90.4			8.4	0.7	0.3	0.2
1100918	1142			12.0											
1100919	0724			11.1											
1100920	1108	6230		11.2	34.4	84.1			71.8			14.8	5.6	7.2	0.6

DATE	TIME	NA	K	CL	BUN	CREAT	eGFR	CA	Mg	P	CRP	ESR	PCT	D-Dimer	LACTATE
1100915	2005	138	4.4		22	1.48	50.39	9.5							
1100916	1451														16.6

1100916	1453	139	4.4		20	1.39	54.17	8.2							
1100920	1059														9.7
1100920	1109	138	4.1		14	1.24	61.8	8.4							

DATE	TIME	GLUCOSE	HbA1C	CHOL	TG	HDL	LDL	KETONE	CK	CKMB	TRO-T	PT-P	APTT-P	NT-pro BNP
1100915	2000											10.3	28.4	
1100915	2002	111												
1100915	2007		6.0											

特殊檢查

病理報告

- 活體組織切片病理報告　手術日期：**110/09/16**　病理號：**S1029395**

診斷醫師:王任卿 報告日期:110/09/24
Pathologic diagnosis:
1. Sigmoid colon, laparoscopic anterior resection --- Status post polypectomy with residual intramucosal adenocarcinoma, moderately differentiated. The bilateral cut ends and mesocolic lymph nodes (0/10) are free of tumor.
2. IMA root, dissection --- Unremarkable lymph node (0/1).
3. Colon, cut end, distal, resection --- Unremarkable.
Ancillary study for diagnosis:
1. Deep cut done for section E.
2. Recut and IHC stain for CD34 (highlights vessels) done for section F.
Prognostic and predictive factor:
(combined with report S1018445)
1. Tumor size: 0.6 cm.
2. Depth of invasion: Subucosa (presented in previous polypectomy specimen, S1018445).
3. Histologic grade: G2: Moderately differentiated.
4. Angiolymphatic invasion: Not identified.
5. Perineural invasion: Not identified.
6. Tumor deposits (discontinuous extramural extension): Not identified.
7. Macroscopic tumor perforation: Not identified.
8. Surgical Margins: a) Proximal Margin: Uninvolved by invasive carcinoma, distance of tumor from margin: 11 cm. b) Distal Margin: Uninvolved by invasive carcinoma, distance of tumor from margin: 3 cm. c) Radial or Mesenteric Margin: Uninvolved by invasive carcinoma, distance of tumor from margin: 4 cm.
9. Lymph node status: Uninvolved (0/11).
10. Extranodal involvement: Not identified.
11. Tumor regression grading S/P CCRT: No known presurgical therapy.
12. Type of polyp in which invasive carcinoma arose: Not identified.
13. Additional pathologic findings: Not identified.
14. Pathological TNM Stage: pT1N0 (According to the eighth edition, American Joint Committee on Cancer Staging Guidelines for Tumors).
15. TNM descriptors: Not applicable.

Gross description:
The specimen consists of 1) a segment of colon, 15 cm in length and 5 cm in proximal cut end circumference, 6 cm in distal circumference, and mesocolic soft tissue. A gray tan soft polypoid tumor, 0.7x0.7 cm, is noted, 3 cm to the distal cut end. On cross section, the tumor invades to the submucosa with 4 cm to the radial margin. The both cut ends are free of tumor grossly. 2) IMA root, a piece of tan yellow soft tissue, 4x3x2 cm. 3) distal cut end, a piece of tan mucosa-covered soft tissue, 3x2x1 cm. Representative part for section: A) specimen 2 B) specimen 3 C) proximal cut end D) radial margin E) mucosa F-G) tumor H-K) lymph node.
Note:
This case has been peer reviewed by two doctors.
#T-59470_2 #M-81403_2 2 1105 B0000F
#T-C4560_2 #M-09183_2 0 1105 000000

其它

出院時情況

- 3.改門診治療

出院指示

出院計劃

- 門診日期
    - date on 2021/09/28

主治醫師 2062F 蔣鋒帆 / 住院醫師 2336L 洪浚嚴

正式摘要**(電子簽章：000000226198769)**

W52 / W52

doctorRank:B
